# P-670. Absenteeism due to Viral Acute Respiratory Infections in Children, 2024-2025

**DOI:** 10.1093/ofid/ofaf695.883

**Published:** 2026-01-11

**Authors:** Huong Q Nguyen, Jennifer P King, Gigi Zheng, Oluwakemi Alonge, Emma Viscidi, Chelsea Canan, Catherine A Panozzo, Darshan Mehta, Wen-Hsing Wu, Meng Wang, Jennifer K Meece, Evan J Anderson, Joshua Petrie

**Affiliations:** Marshfield Clinic Research Institute, Marshfield, WI; Marshfield Clinic Research Institute, Marshfield, WI; Moderna, Inc., Cambridge, Massachusetts; Marshfield Clinic Research Institute, Marshfield, WI; Moderna Therapeutics, Cambridge, Massachusetts; Moderna, Inc., Cambridge, Massachusetts; Moderna, Inc., Cambridge, Massachusetts; Moderna, Inc., Cambridge, Massachusetts; Moderna, Inc., Cambridge, Massachusetts; Moderna, Inc., Cambridge, Massachusetts; Marshfield Clinic Research Institute, Marshfield, WI; Moderna, Inc., Cambridge, Massachusetts; Marshfield Clinic Research Institute, Marshfield, WI

## Abstract

**Background:**

Acute respiratory illnesses (ARIs) are a common reason for daycare or school absence in children. We assessed virus-specific absenteeism due to ARIs in children enrolled in a medically attended surveillance study and a community cohort.

**Methods:**

ARIs were identified in children (< 18 years) seeking medical care for ARIs (6/1/2024-3/18/2025) or from a community cohort (9/29/2024-4/3/2025). Adenovirus, influenza, seasonal coronavirus (sCoV), human metapneumovirus, parainfluenza virus, rhinovirus/enterovirus (RV/EV), RSV, and SARS-CoV-2 were detected from respiratory swabs by RT-PCR. Surveys completed approximately 2 weeks after an ARI assessed absenteeism: total missed days (from daycare/school for infected children and from work for household members)/total infections. Viral coinfections were excluded.

**Results:**

Among 363 infected < 5-year-olds, 38% had household members miss ≥ 1 workday due to the child’s viral ARI. 69% of daycare attendees (137/198) missed ≥ 1 day. 28%, 17%, and 17% of attendees were absent due to RV/EV, RSV, and influenza, contributing to 22%, 22%, and 18% of total daycare days missed, respectively. Daycare absenteeism ranged from 1.5 days/ARI for RV/EV to 3.5 days/ARI for RSV.

Among 209 infected 5-12-year-olds, 42% had household members miss ≥ 1 workday due to the child’s viral ARI. 70% of school attendees 5-12 years (130/185) missed ≥ 1 day. 37% and 35% of children were absent due to influenza and RV/EV, contributing to 45% and 32% of total school days missed, respectively. School absenteeism ranged from 1.0 days/ARI for sCoV to 3.6 days/ARI for influenza.

Among 131 infected 13-17-year-olds, 23% had household members miss ≥ 1 workday due to the child’s viral ARI. 69% of school attendees 13-17 years (75/108) missed ≥ 1 day. 40% and 33% of children were absent due to RV/EV and influenza, contributing to 41% and 34% of total school days missed, respectively. School absenteeism ranged from 0.8 days/ARI for sCoV to 2.3 days/ARI for influenza.

Absenteeism was generally higher for children who sought care vs no care for their viral ARI.

**Conclusion:**

In 2024-2025, approximately 70% of children with ARI across all ages missed ≥1 day of daycare or school. Viral ARIs in younger children resulted in higher work absenteeism by household members than viral ARIs in older children.

**Disclosures:**

Huong Q. Nguyen, PhD, MPH, CSL Seqirus: Advisor/Consultant|CSL Seqirus: Grant/Research Support|GSK: Grant/Research Support|ModernaTX: Advisor/Consultant|ModernaTX: Grant/Research Support Jennifer P. King, MPH, GSK: Grant/Research Support|ModernaTX, Inc.: Grant/Research Support Gigi Zheng, MD, PhD, ModernaTX: Employee|ModernaTX: Stocks/Bonds (Public Company) Oluwakemi Alonge, MPH, CPH, CSL Seqirus: Grant/Research Support|GSK: Grant/Research Support|ModernaTX: Grant/Research Support Emma Viscidi, PhD, MHS, ModernaTX: Employee|ModernaTX: Stocks/Bonds (Public Company) Chelsea Canan, PhD, ModernaTX: Employee|ModernaTX: Stocks/Bonds (Public Company) Catherine A. Panozzo, PhD, ModernaTX: Employee|ModernaTX: Stocks/Bonds (Public Company) Darshan Mehta, PhD, ModernaTX: Employee|ModernaTX: Stocks/Bonds (Public Company) Wen-Hsing Wu, MS, Moderna: Stocks/Bonds (Public Company) Meng Wang, PhD, ModernaTX: Employee|ModernaTX: Stocks/Bonds (Public Company) Jennifer K. Meece, PhD, CSL Seqirus: Grant/Research Support|GSK: Grant/Research Support|ModernaTX: Grant/Research Support Evan J. Anderson, MD, Moderna: Stocks/Bonds (Public Company) Joshua Petrie, PhD, CSL Seqirus: Advisor/Consultant|CSL Seqirus: Grant/Research Support|ModernaTX: Grant/Research Support

